# EphA2 Interacts with Tim-4 through Association between Its FN3 Domain and the IgV Domain of Tim-4

**DOI:** 10.3390/cells10061290

**Published:** 2021-05-22

**Authors:** Byeongjin Moon, Susumin Yang, Kwangwoo Kim, Juyeon Lee, Dongtak Jeong, Daeho Park

**Affiliations:** 1 School of Life Sciences, Gwangju Institute of Science and Technology, Gwangju 61005, Korea; byeongjinmoon@gist.ac.kr (B.M.); susuminy@gist.ac.kr (S.Y.); wooya1127@naver.com (K.K.); iris260@gist.ac.kr (J.L.); 2 Center for Cell Mechanobiology, Gwangju Institute of Science and Technology, Gwangju 61005, Korea; 3 Department of Molecular and Life Science, College of Science and Convergence Technology, Hanyang University ERICA Campus, Ansan 15588, Korea; cooljdt@hanyang.ac.kr

**Keywords:** Tim-4, EphA2, interaction, efferocytosis, IgV domain, FN3 domain

## Abstract

Tim-4 promotes the engulfment of apoptotic cells or exogenous particles by securing them on phagocytes. It is unable to transduce signals by itself but helps other engulfment receptors sense and internalize them. However, the identity of the engulfment receptors collaborating with Tim-4 is still incompletely understood. In this study, we searched for a candidate transmembrane protein with a FN3 domain, important for interaction with Tim-4, in silico and investigated whether it indeed interacts with Tim-4 and is involved in Tim-4-mediated phagocytosis. We found that EphA2 containing a FN3 domain in the extracellular region interacted with Tim-4, which was mediated by the IgV domain of Tim-4 and the FN3 domain of EphA2. Nevertheless, we found that EphA2 expression failed to alter Tim-4-mediated phagocytosis of apoptotic cells or polystyrene beads. Taken together, our findings suggest that EphA2, a new Tim-4 interacting protein, may intervene in a Tim-4-mediated cellular event even if it is not phagocytosis of endogenous or exogenous particles and vice versa.

## 1. Introduction

Apoptotic cells generated in multicellular organisms are swiftly and continuously removed, which is necessary during development and for tissue homeostasis [1]. A crucial step in this essential cellular event, also called efferocytosis, is the recognition of apoptotic cells by phagocytes, which is mediated by the interaction between ligands specifically exposed on apoptotic cells and their receptors on phagocytes [2]. A well-known ligand in apoptotic cells is phosphatidylserine (PS), which is located in the cytosolic side of the plasma membrane in normal cells but is exposed on the surface of cells undergoing apoptosis [3]. The PS on apoptotic cells is recognized by phagocytes in two different ways. PS is directly sensed by PS receptors on phagocytes or alternatively recognized through bridging molecules linking PS to receptors on phagocytes [4,5,6].

One of the best characterized PS receptors is Tim-4. It binds to PS on apoptotic cells and promotes efferocytosis [7,8]. It is thought that the cytoplasmic tail and transmembrane domain of Tim-4 are not essential for Tim-4-mediated efferocytosis since the Tim-4 truncation mutants that lack them are capable of promoting efferocytosis. Thus, Tim-4 is a tethering receptor that secures apoptotic cells on phagocytes, which helps other engulfment receptors sense and phagocytose apoptotic cells [7,9,10]. Due to this notion, how Tim-4 coordinates with the signaling of other engulfment receptors in efferocytosis has been studied over the past decade. Eventually, several proteins, including Fn1, Mertk, and integrins have been identified and are involved in Tim-4-mediated efferocytosis by scaffolding engulfment receptors or transducing signals for recognition of apoptotic cells into phagocytes through biochemical interaction with Tim-4 [11,12,13,14,15,16,17]. The IgV domain of the extracellular region of Tim-4 and a fibronectin type III (FN3) domain of the proteins are essential for the interaction. For example, Fn1 and Mertk possess FN3 domains and the IgV domain of Tim-4 binds to their FN3 domains, resulting in association of Tim-4 with them [13].

There are a variety of transmembrane proteins containing a FN3 domain in their extracellular region. However, it has not been tested whether they interact with Tim-4 and, if any, they are involved in Tim-4 mediated cellular events such as efferocytosis.

In this study, we search for transmembrane proteins possessing a FN3 domain in the extracellular region using the UniProt database and selected EphA2 and Cdon as candidate transmembrane proteins based on the similarity of the expression profile between them and Tim-4. Even though EphA2 and Cdon possess FN3 domains in the extracellular region, only EphA2 interacted and colocalized with Tim-4. As expected, this interaction was mediated by the IgV domain of Tim-4 and the FN3 domain of EphA2, which was confirmed in mammalian cells and yeast cells. Nevertheless, EphA2 expression unaltered Tim-4-mediated phagocytosis of apoptotic cells or exogenous particles such as polystyrene beads. Taken together, we report EphA2 as a Tim-4 interacting protein, which implies the possibility that EphA2 and Tim-4 may intervene with each other in certain cellular events.

## 2. Materials and Methods

### 2.1. Cell Culture and Transfection

293T and LR73 cells were maintained in DMEM (Dulbecco’s Modified Eagle’s Medium) and α-MEM (Alpha’s Modified Eagle’s Medium) supplemented with 10% FBS (Fetal Bovine Serum) and 1% PSQ (Penicillin-Streptomycin-Glutamine), respectively. 293T cells were transfected with calcium phosphate (Promega, Madison, WI, USA), and LR73 cells were transfected with Lipofectamin 2000 (Invitrogen, Waltham, MA, USA).

### 2.2. Plasmids and Antibodies

All expression vectors used in this study were constructed by a PCR-based cloning strategy and sequenced to confirm their fidelity. EphA2 was a gift from Dr. Hironori Katoh and mouse Cdon cDNA was purchased from Open Biosystem (MMM1013-211693000). Tim-4 constructs were previously reported [18]. Cdon was cloned into pEBB, and the ligand-binding domain and fibronectin type III domain of EphA2 was introduced into pVP16. Anti-FLAG (F1804, Sigma Aldrich, St. Louis, MO, USA), anti-GST (SC-138, Santa Cruz Biotechnology, Dallas, TX, USA), anti-HA (#3724, Cell Signaling Technology, Danvers, MA, USA), anti-Myc (SC-40, Santa Cruz Biotechnology, Dallas, TX, USA), anti-EphA2 (05-480, Millipore, Billerica, MA, USA), anti-Tim-4 (SC-79143, Santa Cruz Biotechnology, Dallas, TX, USA), and normal mouse IgG control (MAB002, R&D Systems, Minneapolis, MN, USA) were purchased. Fluorochrome-conjugated secondary antibodies were purchased from Thermo Fisher Scientific (A-11029 and A-11037, Carlsbad, CA, USA).

### 2.3. Immunoprecipitation and Immunoblotting

293T cells transfected with the indicated plasmids were lysed and incubated with appropriate antibodies with protein A/G-conjugated agarose beads (10001D and 10003D, Thermo Fisher Scientific, Carlsbad, CA, USA) at 4 °C for 2 h. Anti-FLAG antibody-conjugated agarose beads (A2220, Sigma Aldrich, St. Louis, MO, USA), anti-HA antibody-conjugated agarose beads (26181, Pierce, Rockford, IL, USA), or glutathione agarose beads (17-0756-01, GE Healthcare, Chicago, IL, USA) were also used to precipitate FLAG-, HA-, or GST-tagged proteins, respectively. Bound proteins on beads were separated by SDS-PAGE, transferred onto nitrocellulose membrane, and detected with appropriate antibodies. The lymph nodes were acquired from the axillary, brachial, and inguinal lymph node of 8-week-old C57/Bl6 mice. Then, the cell clumps were gently dissociated with a 5-mL syringe piston and sieved to isolate individual lymph node cells. The separated cells were lysed with lysis buffer, and then an immunoprecipitation assay was performed using an anti-EphA2 (05-480, Millipore, Billerica, MA, USA).

### 2.4. Immunostaining

LR73 cells cultured on 18 mm Φ coverslips coated with poly-D-lysine were transfected with the appropriate plasmids. One day after transfection, the cells were washed with PBS, fixed with 4% paraformaldehyde in PBS for 15 min, and blocked with 10% BSA in PBS for 30 min. After that, the cells were incubated with anti-HA, anti-Myc, and anti-FLAG primary antibodies in 3% BSA in PBS at 4 °C overnight and then incubated with Alexa fluor 488 and 594 conjugated secondary antibodies for 1 h. After that, the cells were stained by Hoechst 33342 (H1399, Invitrogen, Eugene, OR, USA). Images were taken by confocal microscopy (FV1000 SPD, Olympus, Tokyo, Japan).

### 2.5. Proximity Ligation Assay

Proximity ligation assay was performed according to the manufacturer’s protocol (DUO92101, Sigma Aldrich, St. Louis, MO, USA). Briefly, LR73 cells transfected with the indicated plasmids were fixed with 4% paraformaldehyde, blocked with blocking solution for 1 h, and incubated overnight with anti-HA and anti-Myc antibodies at 4 °C. Then, the cells were incubated with anti-mouse and anti-rabbit PLA probes for ligation and amplification in appropriate buffers and fixed with Duolink^®^ In Situ Mounting Media with DAPI. Images were acquired by using a confocal microscope (FV1000).

### 2.6. Efferocytosis Assay

Efferocytosis assays were previously described [19]. Briefly, LR73 cells transfected with the indicated plasmids were incubated with TAMRA-labeled apoptotic thymocytes or red-fluorescent polystyrene beads (F8826, Invitrogen, Eugene, OR, USA) at 37 °C for 2 h. Then, the cells were extensively washed with ice-cold PBS, trypsinized, and analyzed by flow cytometry (BD, FACS Canto II). Raw data from acquired flow cytometry were analyzed by FLOWJO software (FlowJo LLC, Ashland, OR, USA).

### 2.7. Yeast Two-Hybrid Assay

Yeast two-hybrid assay was previously described [19]. Briefly, HF7C cells were transformed with bait and prey plasmids by the LiAc-based method, and plated on the non-selective plate lacking Trp and Leu. The cells growing on the plate were transferred to liquid media lacking Trp and Leu and cultured overnight. After that, the cells were serially diluted and dotted on the selective plate containing 5 mM 3-amino-1,2,4-triazole (3-AT) with His, Trp, and Leu.

### 2.8. Statistical Analysis

All data were shown as mean ± standard deviation. Each experiment was repeated at least three times independently. Data were analyzed by one-way ANOVA using the GraphPad Prism 7 software (Prism 7, GraphPad Software, La Jolla, CA, USA). It was considered that differences were statistically significant when the *p*-values were less than 0.05.

## 3. Results

### 3.1. EphA2 Interacts with Tim-4

**Figure 1:**
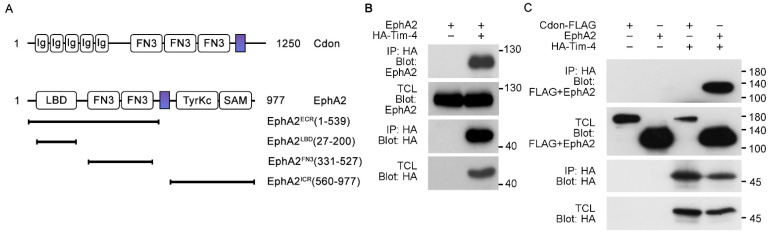
Identification of Tim-4 interacting proteins (**A**) Schematic diagram of Cdon and EphA2 constructs used in the study. The colored rectangles indicate transmembrane domains; Ig, immunoglobulin domain; LBD, ligand-binding domain; FN3, fibronectin type III domain; TyrKc, tyrosine kinase domain; SAM, sterile alpha motif; ECR, extracellular region; ICR, intracellular region. (**B**,**C**) 293T cells transfected with the indicated plasmids were lysed, and the lysates were incubated with anti-HA antibody-conjugated agarose beads. Bound proteins on beads were separated by SDS-PAGE and detected with the indicated antibodies. IP, immunoprecipitation; TCL, total cell lysates.

Some of the Tim-4 interacting proteins identified contain the fibronectin type III (FN3) domain, which mediates their interaction with Tim-4 by binding to the IgV domain of Tim-4 [11,13]. These Tim-4 interacting proteins play important roles in the signaling of Tim-4-mediated efferocytosis. In order to validate whether transmembrane proteins containing a FN3 domain are able to interact with Tim-4 and involved in signaling in Tim-4-mediated efferocytosis, we first searched for transmembrane proteins containing a FN3 domain using the UniProt database and selected candidate transmembrane proteins likely interacting with Tim-4 based on the similarity of their expression profile to that of Tim-4, which resulted in Cdon and EphA2 (Figure 1A and Supplementary Table S1). We then tested whether the candidates are proteins interacting with Tim-4. To do this, HA-tagged Tim-4 was overexpressed with Cdon or EphA2 in 293T cells and immunoprecipitated with an anti-HA antibody. EphA2 was appreciably co-precipitated with Tim-4 (Figure 1B), but Cdon failed to be co-precipitated with Tim-4 (Figure 1C), indicating that Tim-4 interacts with EphA2.

Next, we asked whether the interaction occurs in situ using two different approaches.

**Figure 2:**
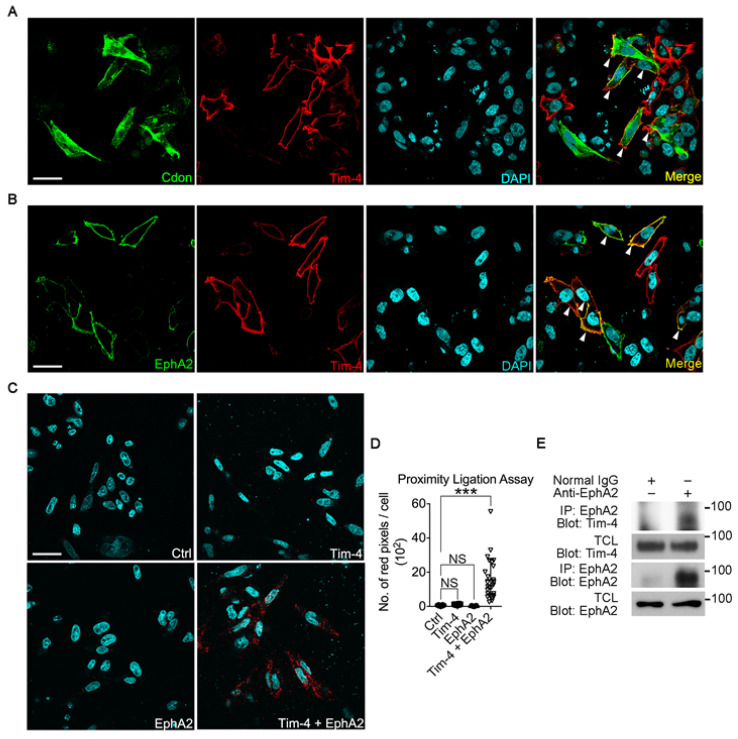
EphA2 interacts with Tim-4 in situ (**A**,**B**) LR73 cells transfected with EphA2, Tim-4, or both EphA2 and Tim-4 were incubated with anti-HA (Tim-4) and anti-FLAG (Cdon, A) or anti-EphA2 (**B**) antibodies and then stained with Alexa Fluor 488- and Alexa Fluor 594-conjugated secondary antibodies. Images were acquired by confocal microscopy. Arrowheads indicate cells expressing both Tim-4 and EphA2 (**A**) or Cdon (**B**). Scale bar, 20 µm. (**C**,**D**) A proximity ligation assay was performed. LR73 cells transfected with the indicated plasmids were fixed, blocked, and incubated with anti-HA and anti-EphA2 antibodies. Then, the cells were incubated with the amplification solution at 37 °C overnight. Images were acquired by confocal microscopy (**C**) and quantified (**D**). Scale bar, 20 µm. Data are shown as the mean ± standard deviation. NS, not significant. *** *p* < 0.001. (**E**) Lymph nodes from mice were lysed, and the lysates were incubated with an anti-EphA2 antibody and protein A/G agarose beads. Bound proteins were detected with the indicated antibodies.

To validate this, first, Tim-4 and the candidates were expressed in LR73 cells, and their co-localization with Tim-4 was tested. EphA2 and Tim-4 were distinctively expressed along the cell boundary, indicating that they mainly localized to the plasma membrane while Cdon localized to the plasma membrane and cytosol. In addition, only the fluorescent signals from EphA2 were noticeably superimposed with those from Tim-4 but not Cdon (Figure 2A,B). Second, we performed a proximity ligation assay (PLA), a tool allowing in situ detection of protein-protein interaction. Only cells expressing both Tim-4 and EphA2 showed PLA signals, but not cells only expressing Tim-4 or EphA2 (Figure 2C,D). We next tested whether Tim-4 interacts with EphA2 at physiological expression levels. Both Tim-4 and EphA2 were expressed in lymph nodes (Figure 2E). Thus, the interaction between EphA2 and Tim-4 at endogenous protein levels was tested in the tissue. Co-precipitated Tim-4 with EphA2 was detectable as EphA2 was immunoprecipitated by an anti-EphA2 antibody (Figure 2E). These data indicate that not all transmembrane proteins containing a FN3 domain interact with Tim-4; however, but the EphA2 protein interacts with Tim-4.

### 3.2. The Extracellular Regions Mediate Interaction between EphA2 and Tim-4

**Figure 3:**
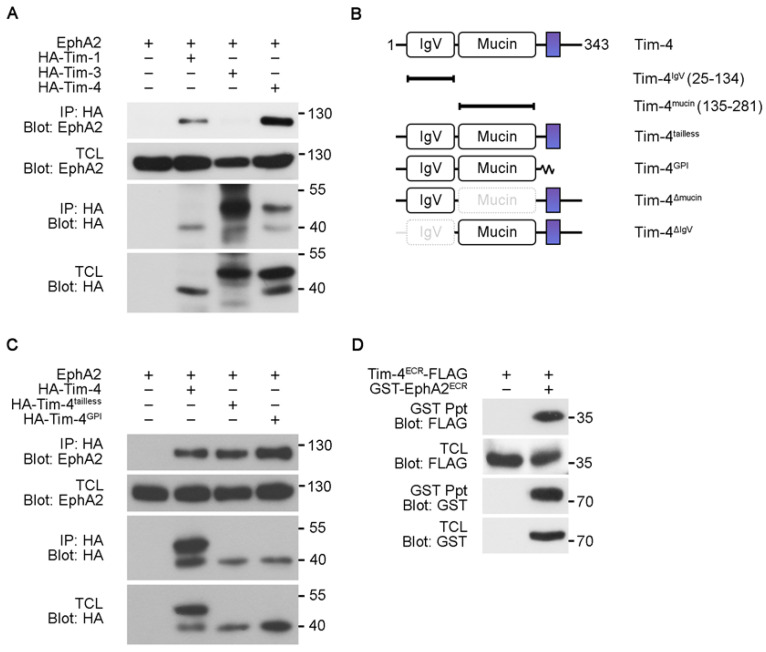
The extracellular regions of EphA2 and Tim-4 mediate their interaction (**A**) 293T cells transfected with the indicated plasmids were lysed two days after transfection. Then the lysates were incubated with anti-HA antibody-conjugated agarose beads, and bound proteins were detected with the indicated antibodies. (**B**) Schematic diagram of Tim-4 mutants used in the study. The colored rectangles indicate transmembrane domains; GPI, glycophosphatidylinositol. (**C**,**D**) 293T cells transfected with the indicated plasmids were lysed, and then the lysates were incubated with anti-HA antibody-conjugated antibody (**C**) or glutathione agarose beads (**D**). Bound proteins were detected by immunoblotting. Ppt, precipitation.

Three of the Tim gene family members, Tim-1, Tim-3, and Tim-4, are conserved between mouse and human [9]. They all recognize phosphatidylserine on apoptotic cells and are involved in efferocytosis, phagocytosis of apoptotic cells [8,20,21]. We thus tested whether the homologs also interact with EphA2 using co-immunoprecipitation assay. EphA2 was co-precipitated with Tim-4 or Tim-1, but it was not co-precipitated with Tim-3 (Figure 3A), indicating that Tim-1 as well as Tim-4 interact with EphA2. We next investigated which regions of the proteins mediate the interaction. We used Tim-4 truncation mutants, Tim-4^tailless^ and Tim-4^GPI^ lacking the cytoplasmic tail and both the transmembrane region and the cytoplasmic tail, respectively, to attain an initial clue about regions mediating the interaction (Figure 3B). Both mutants interacted with EphA2 as robustly as full-length Tim-4 (Figure 3C), suggesting that the cytoplasmic tail and transmembrane domain of Tim-4 are unnecessary for its interaction with EphA2. Then, we further tested whether the extracellular regions of EphA2 and Tim-4 certainly interact with each other. To do this, the extracellular regions of EphA2 and Tim-4 were expressed in 293T cells and the extracellular region of EphA2 was precipitated with glutathione sepharose-conjugated beads. The extracellular region of Tim-4 was appreciably co-precipitated with that of EphA2 (Figure 3D), indicating that EphA2 and Tim-4 interact through their extracellular regions.

### 3.3. The IgV Domain of Tim-4 Is Necessary for Tim-4-EphA2 Interaction

**Figure 4:**
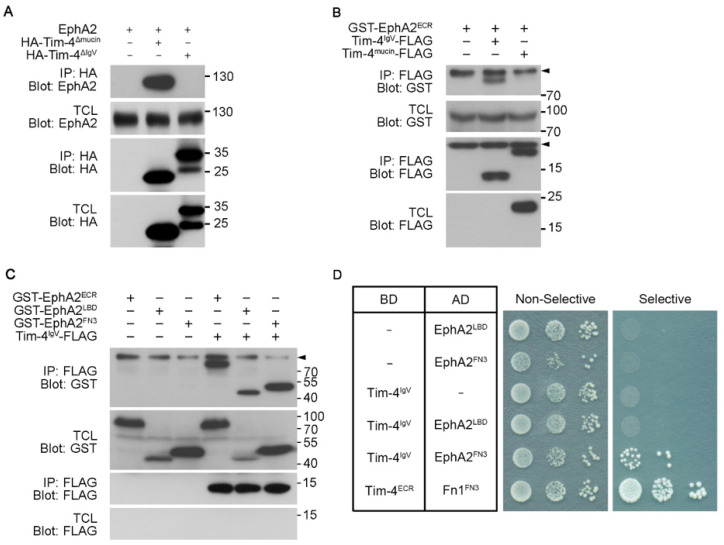
The IgV and FN3 domains are crucial for the interaction of Tim-4 with EphA2 (**A**–**C**) 293T cells were transfected with the indicated plasmids. Two days after transfection, the cells were lysed, and the lysates were incubated with anti-HA antibody-(**A**) or anti-FLAG antibody-conjugated agarose beads (**B**,**C**). Then, the bead-bound proteins were separated by SDS-PAGE and detected with the indicated antibodies. Arrowheads indicate nonspecific bands. Note that Tim-4^IgV^-FLAG was not detectable in TCL. (**D**) Yeast cells transformed with the indicated plasmids were dotted on selective or non-selective media. Cells on the non-selective media were used to show the number of cells dotted. Yeast transformants expressing both Tim-4^ECR^ and Fn1^FN3^ were used as a positive control. BD, binding domain; AD, activation domain.

Next, we dissected which domains in the extracellular regions of EphA2 and Tim-4 are important for the interaction. Interaction between EphA2 and Tim-4 deletion mutants, Tim-4^ΔIgV^ and Tim-4^Δmucin^, was first evaluated (Figure 3B). EphA2 was co-precipitated with Tim-4^Δmucin^ but not with Tim-4^ΔIgV^ (Figure 4A), suggesting that the IgV domain of Tim-4 is necessary for the interaction between Tim-4 and EphA2. Additionally, the IgV domain of Tim-4 was also able to precipitate the extracellular region of EphA2. In contrast, the mucin domain of Tim-4 was unable to precipitate it (Figure 4B), which supports that the IgV domain mediates the interaction with EphA2. We next asked which domain in the extracellular region of EphA2 interacts with the IgV domain. Similarly, Tim-4^IgV^ and EphA2 truncation mutants, EphA2^ECR^, EphA2^LBD^, and EphA^FN3^, were expressed in 293T cells and immunoprecipitation assay was performed. As expected, EphA2^FN3^ was co-precipitated with Tim-4^IgV^, but unexpectedly, the EphA2^LBD^ was also co-precipitated with Tim-4^IgV^ (Figure 4C). In addition, this interaction was not observed when Tim-4^mucin^ was used instead (data not shown). 

Yeast does not express a homolog of either EphA2 or Tim-4. Thus, using a yeast two-hybrid assay, we validated whether the interaction was direct or not. Yeast transformants expressing Tim-4^IgV^ and EphA2^FN3^ grew on the selective plate, whereas other transformants expressing truncation mutants of Tim-4 and EphA2 or control vector failed to grow on the selective plate. Especially, the growth of yeast transformed with Tim-4^IgV^ and EphA2^LBD^ was not observed (Figure 4D), suggesting that the interaction between Tim-4 and EphA2 was mediated by the IgV domain of Tim-4 and the FN3 domains of EphA2.

### 3.4. EphA2 Is Not Involved in Tim-4-Mediated Efferocytosis

**Figure 5:**
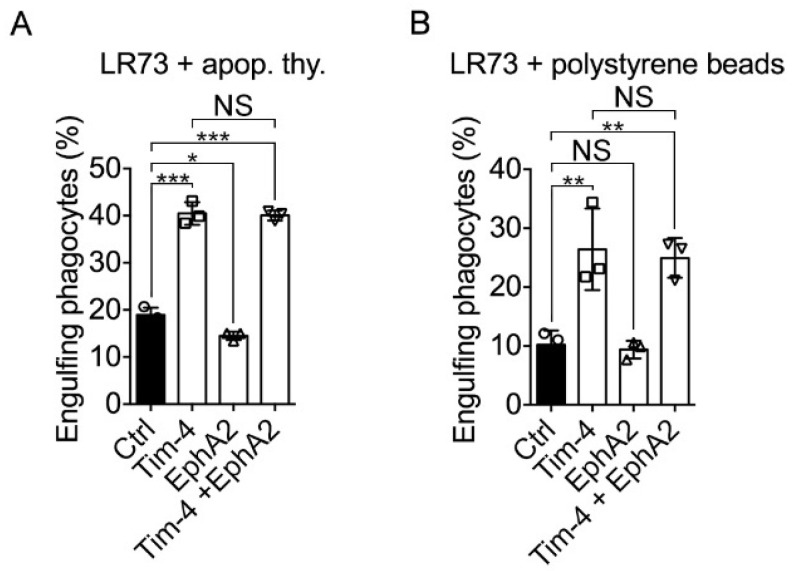
EphA2 unalters Tim-4-mediated phagocytosis (**A**,**B**) Efferocytosis assay was performed using LR73 cells. TAMRA-labeled apoptotic thymocytes (**A**) or red-fluorescent polystyrene beads (**B**) were added to LR73 cells transfected with the indicated plasmids and GFP and incubated for 2. Then, the cells were extensively washed with ice-cold PBS, trypsinized, and analyzed by flow cytometry. Double positive cells for green and red fluorescence were considered as engulfing phagocytes. Data are shown as the mean ± standard deviation. NS, not significant. * *p* < 0.05, ** *p* < 0.01, *** *p* < 0.001.

Tim-4 is known as a tethering receptor binding to PS on apoptotic cells and securing apoptotic cells on phagocytes, which promotes the sensing of apoptotic cells and engulf apoptotic cells by other engulfment receptors [10,11,12,13,15]. In addition, EphA2 may also be involved in efferocytosis because Ephexin4, an EphA2 interacting protein, functions as a GEF for RhoG, which is involved in efferocytosis [22,23,24,25]. Therefore, it is conceivable that EphA2 mediates signaling for Tim-4-mediated efferocytosis. To test this, Tim-4 and EphA2 were expressed in LR73 cells, and the effect of EphA2 expression on Tim-4-mediated efferocytosis was measured. EphA2 expression alone in LR73 cells slightly but significantly inhibited engulfment of apoptotic cells as measured by a percentage of cells engulfing apoptotic cells, but Tim-4 expression prominently promoted engulfment of apoptotic cells as reported previously. However, the engulfment of apoptotic cells by LR73 cells expressing both EphA2 and Tim-4 was similar to that by LR73 cells expressing only Tim-4 (Figure 5A), suggesting that EphA2 is unrelated to Tim-4-mediated efferocytosis. It was also reported that Tim-4 functions as a receptor for not only apoptotic cells but also other exogenous particles such as polystyrene beads and *E. coli* particles [18]. Thus, we also evaluated the effect of EphA2 on Tim-4-mediated phagocytosis of styrene beads. Although phagocytosis of the beads by LR73 cells expressing Tim-4 was superior to that by control cells, phagocytosis of the beads by LR73 cells expressing both Tim-4 and EphA2 was comparable with that by LR73 cells expressing Tim-4 alone (Figure 5B), implying that EphA2 is not involved in Tim-4-mediated phagocytosis.

## 4. Discussion

Originally, Tim-4 was reported as a ligand for Tim-1 regulating T-cell proliferation, but its role as a phosphatidylserine receptor has received more attention [26]. As the cytoplasmic tail and transmembrane domain are dispensable for Tim-4-mediated efferocytosis, it has been believed that a co-receptor transducing signals into phagocytes instead of Tim-4 exits. Initially, two-step engulfment, where Tim-4 secures apoptotic cells, and other engulfment receptors phagocytose them, was suggested [12]. Later several studies showed that Tim-4 directly interacts with Fn1, Mertk, or integrins, to which Tim-4 relays signals during efferocytosis [11,13,14]. However, the limited number of Tim-4 interacting proteins, which help to understand signaling for Tim-4-mediated cellular events, is identified. Due to the necessity of a FN3 domain for interaction with Tim-4, we investigated the possibility that a protein possessing a FN3 domain interacts with Tim-4 and is engaged in Tim-4-mediated efferocytosis. Cdon and EphA2 were final candidates, but only EphA2 interacted with Tim-4. These suggest that a FN3 domain of a transmembrane protein is necessary but not sufficient for interaction with Tim-4. Thus, the three-dimensional structure of a protein may affect interactions with Tim-4.

Previously, it was reported that EphA2 interacts with Ephexin4, a GEF (guanine nucleotide exchange factor) for RhoG, which activates Rac1 and thus promotes the engulfment of apoptotic cells [22,23]. This suggests that EphA2 and Tim-4 have a significant correlation in terms of efferocytosis. Moreover, the observation about the interaction between EphA2 and Tim-4 strengthened the engagement of EphA2 to Tim-4-mediated efferocytosis. However, EphA2 is unlikely involved in Tim-4-mediated efferocytosis, although it interacts with Tim-4. Nevertheless, our findings still provide insights to better understand signaling for Tim-4- or EphA2-mediated cellular events. EphA2 may be engaged in other Tim-4-mediated cellular events such as T-cell proliferation [27,28,29]. EphA2 is involved in various biological processes such as cell migration, proliferation, and differentiation [30,31]. Thus, inversely, Tim-4 may intervene in those EphA2-mediated processes. In these aspects, future studies on the EphA2-Tim-4 interaction will be of interest.

## Data Availability

Data supporting the findings of the study are available within the article and Supplementary materials or from the corresponding author upon reasonable request.

## Supplementary Materials

The following are available online at https://www.mdpi.com/article/10.3390/cells10061290/s1, Table S1: Expression profile of transmembrane proteins containing a FN3 domain.
